# Human-Centered AI Metacognition: Development and Validation of a Scale for Preserving Cognitive Autonomy in Human–AI Collaboration

**DOI:** 10.3390/jintelligence14100270

**Published:** 2026-10-20

**Authors:** Huanhua Lu, Ruihan Chen, Ran Wei

**Affiliations:** 1 School of Marxism, China University of Geosciences (Beijing), Beijing 100083, China; huanhua137117@126.com (H.L.);; 2 Beijing Haidian Association for Science Popularization Education, Beijing 100089, China

**Keywords:** Human-Centered AI Metacognition, cognitive autonomy, metacognition, AI literacy, autonomous cognitive offloading

## Abstract

The rapid advancement of generative artificial intelligence (AI) is transforming human cognitive activities and creating a growing need to preserve cognitive autonomy in human–AI interactions. Although recent research has examined metacognition in AI-mediated environments, existing work has primarily focused on improving AI use and human–AI collaboration, with less attention to how individuals regulate the role and extent of AI involvement in their own cognitive activities. To address this gap, the present study introduced Human-Centered AI Metacognition (HC-AIM), defined as the capacity to recognize AI capabilities and limitations, monitor AI-generated information, and regulate the extent to which AI participates in one’s own cognitive activities while maintaining cognitive autonomy. Across three studies, we developed the Chinese version of the Human-Centered AI Metacognition Scale (HC-AIMS) and conducted an initial psychometric evaluation. Study 1 examined the scale structure using exploratory factor analysis and supported a predominantly unidimensional structure with good internal consistency. Study 2 examined this structure using confirmatory factor analysis and provided evidence of its convergent validity through its association with general metacognition. Study 3 further replicated the scale structure and internal consistency and examined its relationships with AI literacy, collaborative AI metacognition, and autonomous cognitive offloading. HC-AIM was positively associated with all three constructs. Comparisons of theoretically specified factor models provided preliminary evidence of empirical distinguishability between HC-AIM and both AI literacy and collaborative AI metacognition, despite substantial correlations with these constructs. HC-AIM was also positively associated with autonomous cognitive offloading, providing complementary self-reported criterion-related evidence for its validity. Taken together, the findings suggest that HC-AIM represents a contextualized, human-centered form of metacognitive regulation that is closely related to broader metacognitive and AI-related competencies while specifically emphasizing the regulation of AI participation in human cognition. The Chinese version of the HC-AIMS provides a theoretically grounded starting point for investigating human cognition and cognitive autonomy in AI-mediated environments.

## 1. Introduction

In recent years, the rapid development of generative artificial intelligence (GenAI), exemplified by systems such as ChatGPT, has begun to fundamentally reshape how people engage in cognitive activities. Unlike traditional digital technologies, which have primarily served functions such as information retrieval, computational assistance, and information processing, contemporary AI systems are increasingly capable of generating text, explaining knowledge, performing complex reasoning, supporting decision making, and assisting with creative tasks. As a result, AI is gradually shifting from being an external tool to becoming an important cognitive resource that actively participates in human cognitive activities (Clark & Chalmers, 1998; Hutchins, 1995; Parasuraman & Riley, 1997; Risko & Gilbert, 2016).

The integration of AI into cognitive activities creates substantial opportunities for improving the efficiency and scope of human thinking. However, the increasing involvement of AI in cognitive processes also presents new psychological challenges. First, because AI-generated content is often characterized by fluency, coherence, and apparent plausibility, individuals may accept AI-provided information without sufficiently evaluating its accuracy, thereby increasing the risk of automation bias (Parasuraman & Riley, 1997). Second, when individuals rely extensively on AI to perform analytical, judgment, and problem-solving tasks, they may increasingly transfer to external systems cognitive activities that they would otherwise perform themselves, a phenomenon known as cognitive offloading (Risko & Gilbert, 2016). This raises an important question that extends beyond whether AI can improve task performance: How can individuals make use of AI while retaining an active and autonomous role in their own cognition?

### 1.1. Cognitive Autonomy and Metacognition in AI-Mediated Cognition

Cognitive autonomy is particularly relevant to this question because the increasing availability of intelligent external resources changes the conditions under which individuals exercise independent judgment. Building on research into cognitive autonomy, independent judgment, metacognitive regulation, and cognitive offloading (Risko & Gilbert, 2016; Zhu et al., 2026), cognitive autonomy can be understood as an individual’s capacity to independently evaluate information, form judgments, and regulate their own cognitive processes even when external cognitive resources are available to provide support. In AI-mediated environments, maintaining cognitive autonomy does not necessarily mean avoiding AI or refusing to delegate any cognitive activity. Rather, it concerns retaining the capacity to determine when AI assistance is appropriate, critically evaluate AI-generated information, and regulate the extent to which AI participates in one’s own cognitive activities.

This issue is closely connected to metacognition. Metacognition has long been regarded as an important component of higher-order cognition. Flavell (1979) conceptualized metacognition in terms of individuals’ knowledge and regulation of their own cognitive processes, while subsequent research distinguished metacognitive knowledge from metacognitive regulation, including the monitoring, evaluation, and adjustment of cognitive processes in response to task demands and feedback (Nelson & Narens, 1990; Schraw & Dennison, 1994). Metacognitive regulation allows individuals not only to process information but also to evaluate whether their cognitive strategies are appropriate and modify their cognitive activities when necessary.

Traditional conceptualizations of metacognition, however, have largely focused on situations in which individuals themselves perform the relevant cognitive activities. In such contexts, metacognitive regulation involves questions such as whether one understands a task, whether one’s cognitive strategy is effective, and whether one’s thinking needs to be adjusted. AI-mediated cognition introduces an additional object of regulation. When AI participates in thinking, individuals must also consider whether AI assistance is appropriate, how AI-generated information should be evaluated, and how much influence AI should have on their own cognitive processes. Thus, the increasing involvement of AI does not necessarily imply the emergence of an entirely separate form of metacognition. Rather, it creates a new cognitive context in which existing metacognitive processes must also be applied to the individual’s relationship with an external intelligent system.

From this perspective, an important theoretical question is how metacognitive regulation operates when AI becomes an active participant in cognition. In particular, individuals need to regulate not only their own cognitive processes but also the role and extent of AI involvement in those processes. This autonomy-oriented aspect of metacognitive regulation has received comparatively less systematic attention.

### 1.2. The Development and Limitations of Research on AI Metacognition

As AI has become increasingly integrated into learning, work, and decision making, researchers have begun to extend metacognition theory to AI-mediated environments and develop corresponding measurement tools. For example, previous research has developed a GPT-assisted metacognition scale to assess individuals’ metacognitive activities in AI-assisted learning environments, which emphasizes that learners need to set learning goals, monitor their level of understanding, and adjust their learning strategies to achieve more effective AI-assisted learning (Varghese & Sharma, 2026). Another study has examined metacognitive processes related to prompting and developed scales assessing how individuals design, modify, and optimize prompts (Suriano & Plebe, 2026). In addition, Sidra and Mason (2026) proposed the concept of collaborative AI metacognition, emphasizing that when humans and AI jointly perform a task, individuals need to engage in task planning, process monitoring, and outcome evaluation. This perspective moves beyond viewing AI merely as a tool and instead highlights its collaborative role in human cognitive activities (Sidra & Mason, 2026).

Although the above three studies have substantially advanced understanding of metacognition in AI-mediated contexts, they have primarily examined metacognitive processes from the perspectives of learning with AI, interacting with AI, and collaborating with AI. Accordingly, existing research has addressed questions such as: *How can AI-assisted learning be made more effective? How can users obtain higher-quality AI outputs? How can humans and AI coordinate their cognitive activities more effectively?* These questions are important for understanding effective human–AI interaction. However, an equally important question has received comparatively less explicit attention: How can individuals regulate AI’s involvement in their own cognitive activities while preserving cognitive autonomy?

This question becomes particularly important because effective interaction with AI does not necessarily guarantee autonomous cognition. An individual may be proficient in designing prompts or collaborating effectively with AI, yet still have difficulty recognizing when AI assistance is appropriate, evaluating whether AI-generated information should be accepted or independently verified, or adjusting the extent of AI involvement according to task demands. In other words, being capable of using AI effectively and being capable of regulating AI’s role in one’s own cognition are related but not necessarily equivalent. This distinction suggests the need to examine a more autonomy-oriented aspect of metacognitive regulation in AI-mediated cognition.

### 1.3. Human-Centered AI Metacognition: Metacognitive Regulation of AI Involvement in Human Cognition

Building on the foregoing analysis, the present study introduces the concept of Human-Centered AI Metacognition (HC-AIM) to capture the metacognitive regulation of AI’s involvement in human cognition. We define HC-AIM as the capacity to recognize AI capabilities and limitations, monitor AI-generated information, and regulate the extent to which AI participates in one’s own cognitive activities while maintaining cognitive autonomy.

HC-AIM is grounded in the broader metacognitive processes of awareness, monitoring, evaluation, and regulation, but extends their application to a context in which an AI system can actively participate in cognitive activities. Thus, HC-AIM does not imply that individuals possess an entirely separate metacognitive system when interacting with AI. Rather, it concerns how metacognitive regulation is applied when individuals must determine whether, how, and to what extent AI should participate in their own cognition. The central regulatory target is therefore not simply the quality of AI interaction or the efficiency of AI use, but the individual’s cognitive relationship with AI and the preservation of human cognitive autonomy.

Specifically, this capacity comprises three interrelated processes of cognitive regulation. First, AI awareness refers to individuals’ understanding of the strengths and limitations of AI and their ability to determine which cognitive tasks are appropriate for AI assistance and which require independent cognitive engagement. Second, AI monitoring refers to the active evaluation of the plausibility, accuracy, and reliability of AI-generated content rather than accepting AI outputs without sufficient critical evaluation. Third, AI use regulation refers to individuals’ ability to adjust the extent of AI involvement according to task characteristics and their own cognitive needs. This includes recognizing when additional AI assistance is useful, when AI involvement should be reduced, and when independent cognitive engagement is preferable. These three processes are conceptually interrelated because they collectively address a common regulatory problem: how individuals can determine and regulate the appropriate role of AI in their own cognitive activities while retaining responsibility for cognitive evaluation and judgment. Accordingly, HC-AIM is conceptualized as a unified, autonomy-oriented form of metacognitive regulation in AI-mediated cognition. Importantly, this does not imply that HC-AIM is a clearly independent psychological capacity; rather, it represents a context-specific regulatory focus within the broader metacognitive system.

### 1.4. Conceptual Boundaries of HC-AIM

Although HC-AIM shares important metacognitive foundations with existing AI-related metacognition research, its conceptual emphasis differs in several respects. First, GPT-assisted metacognition primarily concerns the regulation of one’s learning processes when AI is used as a learning resource. HC-AIM is broader in task scope and focuses specifically on regulating AI’s participation in the individual’s own cognitive activities, including decisions about when AI assistance is appropriate and when independent cognitive engagement is preferable.

Second, prompting-related metacognition emphasizes the design, evaluation, and refinement of prompts to obtain useful or high-quality AI outputs. Such skills can facilitate effective interaction with AI, but HC-AIM addresses a prior regulatory question: whether AI should be involved in a particular cognitive activity, how much involvement is appropriate, and whether the resulting AI information should be independently evaluated. Thus, effective prompting may be one component of competent AI use, whereas HC-AIM concerns the regulation of AI involvement itself.

Third, collaborative AI metacognition emphasizes metacognitive processes that occur when humans and AI jointly perform cognitive tasks, including planning, monitoring, and evaluating collaborative performance. HC-AIM shares this collaborative and metacognitive foundation. However, its central emphasis is narrower and more autonomy-oriented: it concerns how individuals regulate the role, extent, and boundaries of AI participation while retaining human responsibility for cognitive evaluation and judgment. In this sense, collaborative AI metacognition and HC-AIM should be regarded as closely related rather than completely independent constructs, with HC-AIM placing greater emphasis on the regulation of AI involvement and cognitive autonomy.

Finally, HC-AIM is conceptually related to AI literacy and AI-use skills but should not be equated with them. AI literacy primarily concerns knowledge and understanding of AI capabilities, limitations, applications, and implications, whereas AI-use skills refer to the ability to operate AI systems effectively. HC-AIM concerns how individuals translate awareness of AI and their own cognitive needs into metacognitive decisions about AI involvement. Thus, AI literacy and AI-use skills may provide important foundations for HC-AIM, while HC-AIM focuses on the regulation of the human–AI cognitive relationship.

Taken together, these distinctions do not imply that HC-AIM is unrelated to existing constructs. Rather, HC-AIM occupies a specific position within the broader network of AI-related cognitive and metacognitive processes. Its defining feature is the autonomy-oriented regulation of AI’s role in human cognition, rather than AI use, AI output optimization, or human–AI collaboration per se.

### 1.5. HC-AIM and Autonomous Cognitive Offloading

An important AI-related behavioral tendency relevant to human-centered AI use concerns how individuals incorporate AI into their own cognitive activities. Cognitive offloading refers broadly to the use of external resources to reduce the cognitive demands placed on internal processing (Risko & Gilbert, 2016). With the increasing cognitive capabilities of generative AI, cognitive offloading to AI can take different forms. Recent research has distinguished autonomous cognitive offloading, in which individuals use AI-generated information or suggestions to support or extend their own thinking while remaining actively engaged in the cognitive process, from more dependent forms of offloading in which greater portions of cognitive work are delegated to AI (Zhu et al., 2026). Autonomous cognitive offloading is therefore particularly relevant to human-centered AI use because it allows individuals to draw on AI as a cognitive resource while retaining their own involvement in evaluating and using AI-generated information.

This AI-related behavioral tendency is theoretically consistent with the regulatory focus of HC-AIM. HC-AIM concerns individuals’ capacity to recognize when AI assistance is appropriate, monitor AI-generated information, and regulate the extent of AI involvement in their own cognitive activities. Such metacognitive regulation may enable individuals to incorporate AI assistance without relinquishing their active role in the cognitive process. Accordingly, individuals with higher HC-AIM would be expected to show greater engagement in autonomous cognitive offloading, as reflected in their use of AI to support their cognition while maintaining personal involvement in cognitive processing. Examining the association between HC-AIM and autonomous cognitive offloading therefore provides a theoretically relevant self-reported criterion for evaluating the validity of the HC-AIM scale. A positive association would provide criterion-related evidence that HC-AIM is reflected not only in individuals’ metacognitive regulation of AI involvement but also in how they incorporate AI into their own cognitive activities.

### 1.6. The Present Study

The increasing integration of AI into human cognition makes it increasingly important to understand not only how individuals can use AI effectively, but also how they can regulate AI’s role in their own cognitive activities. Although existing research has begun to examine AI-assisted metacognition, prompting-related metacognition, and collaborative AI metacognition, a systematic measure specifically focused on the human-centered regulation of AI involvement and the preservation of cognitive autonomy remains limited. The present research therefore develops and validates the Chinese version of the Human-Centered AI Metacognition Scale (HC-AIMS). Across three studies, we examine the psychometric properties and conceptual positioning of HC-AIM.

Study 1 developed the initial item pool and examined the underlying factor structure and internal consistency of the Chinese version of the HC-AIMS using exploratory factor analysis (EFA). The items were designed to capture the interrelated processes of AI awareness, AI monitoring, and AI-use regulation as manifestations of a unified metacognitive capability.

Study 2 used an independent sample to examine the structural stability of the scale through confirmatory factor analysis (CFA) and to investigate its relationship with general metacognitive ability. Because HC-AIM is conceptualized as a context-specific extension of metacognitive regulation, a positive association with general metacognition is expected.

Study 3 further examined the conceptual and nomological position of HC-AIM by assessing its relationships with AI literacy, collaborative AI metacognition, and autonomous cognitive offloading. These analyses were intended to clarify how HC-AIM relates to closely connected AI-related competencies and metacognitive constructs, while also examining its association with the autonomy-oriented outcomes that constitute the central theoretical rationale for the construct.

Taken together, the three studies aimed to provide an initial psychometric and theoretical foundation for understanding HC-AIM as a context-specific, human-centered form of metacognitive regulation. Rather than suggesting that cognitive competence in the age of AI consists simply of greater AI use or AI proficiency, the present research highlighted another dimension of human–AI cognition: the capacity to determine the appropriate role of AI in one’s own cognitive activities while retaining cognitive autonomy.

## 2. Study 1: Item Development and Exploratory Factor Analysis of the HC-AIMS

### 2.1. Purpose of Study 1

Study 1 aimed to develop the Chinese version of the HC-AIMS and to preliminarily examine its factor structure and internal consistency.

### 2.2. Theoretical Operationalization and Item Development of the HC-AIMS

Building on the theoretical conceptualization of HC-AIM presented in the Introduction, the present study further operationalized the construct as a measurable psychological variable. Traditional metacognition theories propose that effective regulation of one’s cognitive processes relies on awareness, monitoring, and regulation of cognition (Flavell, 1979; Nelson & Narens, 1990; Schraw & Dennison, 1994). In the context of AI-mediated cognition, HC-AIM was conceptualized as a context-specific extension of traditional metacognition, referring to individuals’ ability to manage and regulate the ways and the extent to which AI participates in cognitive activities while maintaining their own cognitive autonomy. Specifically, the content domain of HC-AIM was derived from the three interrelated cognitive processes outlined in the Introduction: awareness of AI’s capabilities and limitations, monitoring of AI-generated information, and regulation of the extent to which AI participates in cognitive activities. Importantly, these processes were not conceptualized as three independent psychological dimensions. Rather, they were considered different manifestations of a unified metacognitive capability in human–AI collaborative cognitive environments. In other words, AI awareness, AI monitoring, and AI-use regulation collectively reflect an individual’s overall ability to actively manage AI’s involvement in cognitive activities and maintain individuals’ cognitive autonomy during human–AI collaborative cognition. Accordingly, we expected HC-AIM to exhibit a unidimensional structure, with the different cognitive-regulatory processes representing related aspects of the same underlying capability.

Based on the theoretical framework described above, the research team generated an initial pool of 12 items to capture manifestations of HC-AIM in AI-mediated cognitive activities. Item development was guided by three principles. First, the items were designed to assess how individuals regulate AI’s involvement in cognitive activities rather than their technical proficiency in using AI tools, general AI knowledge, or prompt-engineering skills. Second, the item pool was designed to cover AI awareness, AI monitoring, and AI-use regulation as interrelated processes, thereby representing HC-AIM as a unified construct rather than as three separate dimensions. Third, the items were worded to capture relatively stable cognitive tendencies in AI use rather than behaviors occurring during a specific human–AI interaction.

The initial 12-item pool was reviewed by four experts, including two professors with expertise in psychology and two professors with expertise in AI. The experts evaluated the items with respect to four criteria: (a) whether each item was consistent with the theoretical definition of HC-AIM, (b) whether the item adequately represented the core content of the construct, (c) whether the wording was accurate, clear, and easy to understand, and (d) whether the item could be distinguished from related concepts such as AI literacy and AI skills.

The expert review resulted in the removal of three items. One item (“I understand that I should pay attention to protecting personal privacy and information security when using AI tools.”) was removed because it was considered insufficiently consistent with the theoretical definition of HC-AIM, one (“I will judge whether the content provided by AI is good.”) was removed because its wording was unclear, and one (“I will control myself when using AI.”) was removed because it was considered potentially ambiguous. The remaining nine items were retained for subsequent exploratory factor analysis (EFA) and psychometric evaluation. The expert evaluation was qualitative rather than quantitative. Specifically, the experts did not provide numerical ratings that would permit calculation of formal content-validity indices such as the Content Validity Coefficient (CVC), Aiken’s V, or Lawshe’s Content Validity Ratio (CVR). We therefore did not calculate these indices. Instead, item retention and removal were determined through the expert panel’s qualitative assessment of theoretical relevance, content coverage, clarity, and conceptual distinctiveness.

The HC-AIMS was originally developed and evaluated in Chinese. The expert review was conducted using the Chinese version, and the scale was also administered to participants in Chinese. The English version presented in this manuscript was subsequently translated from the Chinese version for reporting purposes. The initial English translation was prepared by the author team based on the final Chinese items. To enhance linguistic clarity and semantic correspondence with the original Chinese items, the preliminary English translation was reviewed and revised by two professors of English from the same university as the corresponding author. Following their feedback, the author team further discussed and refined the wording and finalized the English translation presented in this article. The English version was therefore translated by the author team and subsequently reviewed and revised by two English-language specialists. Because the English translation was not used for data collection and was not subjected to a formal translation/back-translation procedure or independent psychometric validation, it should not be considered a separately validated English-language version of the HC-AIMS. The Chinese version remains the version empirically validated in the present research.

Before completing the scale, participants received the following standardized instructions: “Please judge how well each of the following statements applies to you based on your actual experience using generative AI tools, and select the corresponding number. There are no right or wrong answers; please respond based on your first impression. Response options: 1 = Strongly Disagree; 2 = Disagree; 3 = Neutral; 4 = Agree; 5 = Strongly Agree.”

### 2.3. Methods

#### 2.3.1. Participants

A total of 210 participants (Sample 1) were recruited using convenience sampling in general education courses at the university. Recruitment was conducted through university classroom settings, and participants completed the questionnaire online rather than using paper-and-pencil questionnaires. Participants ranged in age from 18 to 28 years (*M* = 23.27, *SD* = 1.24), including 72 women and 138 men. All participants had prior experience using generative AI tools, such as ChatGPT, DeepSeek, or Kimi. The frequency of generative AI use was assessed using a five-point scale ranging from very frequent to very infrequent. All participants reported a frequency rating of 4 or higher, indicating that all participants had relatively frequent experience using generative AI tools. All participants voluntarily participated in the study and provided informed consent prior to participation. No monetary or other compensation was provided. Because recruitment was conducted through voluntary classroom-based recruitment and participants independently chose whether to access and complete the online questionnaire, no fixed number of individuals was formally invited to participate. Therefore, a conventional questionnaire response rate could not be calculated. The study was approved by the Institutional Review Board of China University of Geosciences (Beijing) (No. 20251225). Before the main analyses, three screening criteria were applied to all responses. First, questionnaires with missing responses on any item were considered incomplete and excluded. Second, completion time was screened using a reference-based criterion. Ten pilot respondents (graduate students from the corresponding author’s research group) completed the questionnaire, and their mean completion time (M = 63 s) served as the reference. Responses were excluded if completion time was shorter than one-third of this mean (<21 s) or longer than three times this mean (>189 s). Third, duplicate submissions from the same IP address were identified; if multiple responses had originated from the same IP, only the first submission would have been retained. In the present study, no responses were excluded by any of these criteria (incomplete: 0; too short: 0; too long: 0; duplicate IP: 0). All 210 responses were therefore retained for analysis.

#### 2.3.2. Measure

HC-AIM was assessed using the Chinese version of the HC-AIMS developed by the research team. The scale consisted of nine items rated on a 5-point Likert scale ranging from 1 (strongly disagree) to 5 (strongly agree). Higher scores indicated higher levels of HC-AIM.

#### 2.3.3. Data Analysis

EFA was conducted to examine the underlying factor structure of the Chinese version of the HC-AIMS. The internal consistency of the scale was assessed using Cronbach’s alpha coefficient. The initial EFA used principal axis factoring (PAF) under an approach that treated the item responses as continuous. In response to reviewer concerns regarding the ordinal nature of the five-point response format, we additionally conducted parallel analysis and a supplementary PAF based on polychoric correlations to examine the robustness of the factor structure under an ordinal-data framework. These supplementary analyses were used to evaluate whether the primary factor-analytic conclusion was sensitive to the treatment of the response categories.

### 2.4. Results

#### 2.4.1. Exploratory Factor Analysis

First, the suitability of the data for factor analysis was examined using the Kaiser–Meyer–Olkin (KMO) measure and Bartlett’s test of sphericity. The KMO value was 0.939, exceeding the recommended threshold, and Bartlett’s test of sphericity was significant, χ^2^(36) = 1285.552, *p* < .001, indicating that the data were suitable for EFA.

An EFA was first conducted using SPSS 27, with the nine HC-AIMS items treated as continuous variables. Principal axis factoring (PAF) based on Pearson correlation was used to examine the underlying factor structure. As shown in Table 1, all nine items loaded strongly on a single factor, with factor loadings ranging from 0.728 to 0.865. The one-factor solution explained 61.23% of the total variance. These results indicated a clear and coherent one-factor structure, with all items showing substantial loadings on the same underlying factor.

To further evaluate the number of factors to retain, a parallel analysis was subsequently conducted in R using the psych package (Version 2.6.5; R Version 4.5.3). Given that the nine HC-AIMS items were rated on a five-point Likert scale, polychoric correlations were used as the basis for factor extraction. The parallel analysis based on polychoric correlations (100 simulations) suggested a one-factor solution (Table 2). Specifically, the first actual eigenvalue (6.265) substantially exceeded its corresponding simulated mean (0.544) and resampled mean (0.641), whereas the second actual eigenvalue (0.273) was only slightly above the simulated mean (0.241) and below the resampled mean (0.293). This pattern supported the retention of one factor.

As a supplementary analysis, PAF was also conducted in R using the polychoric correlation matrix and oblimin rotation. As shown in Table 3, all nine items loaded strongly on the single factor, with standardized loadings ranging from 0.781 to 0.911. The communalities ranged from 0.61 to 0.83, indicating that the factor accounted for a substantial proportion of variance in each item. The one-factor solution explained 69.61% of the total variance.

Taken together, the primary PAF based on continuous-variable treatment, the parallel analysis based on polychoric correlations, and the polychoric-correlation PAF converged in supporting a unidimensional structure of the HC-AIMS.

#### 2.4.2. Sensitivity Analysis

To examine the robustness of the factor structure to invariant responding, a sensitivity analysis was conducted after excluding 79 participants who selected the same response category across all nine HC-AIMS items, resulting in a reduced sample of 131 participants. In this reduced sample, parallel analysis indicated a two-factor solution, with the second observed eigenvalue (0.499) exceeding both the random simulation mean (0.318) and the resampling simulation mean (0.370).

To further examine the substantive nature of this solution, PAF was conducted in the reduced sample. The PAF yielded a recognizable two-factor loading pattern, with A1–A4 loading primarily on one factor and A5–A9 loading primarily on the other. However, this statistical pattern did not correspond to a coherent theoretical distinction within HC-AIM. Specifically, A1–A3 primarily represent AI awareness, whereas A4 represents AI monitoring; conversely, A5 also represents AI monitoring, whereas A6–A9 primarily represent AI-use regulation. Thus, the two-factor solution separates the two conceptually related monitoring items across different factors while combining different theoretical processes within each factor. Taken together, although the sensitivity analysis identified a statistically recognizable two-factor pattern, the resulting factors did not have a clear and theoretically meaningful interpretation as two distinct dimensions of HC-AIM. Detailed PAF results are reported in the Supplementary Materials.

Importantly, this sensitivity analysis was intended to examine the robustness of the factor-retention results rather than to establish that invariant responders provided invalid data. The full Study 1 sample consistently supported a one-factor solution across the primary and supplementary factor-analytic analyses. The sensitivity analysis indicates that a two-factor statistical pattern can emerge under the reduced-sample condition, but provides limited support for interpreting this pattern as evidence of two substantively distinct dimensions. Accordingly, we interpret the HC-AIMS as predominantly or substantively unidimensional while acknowledging that secondary factor patterns may emerge under particular sample conditions.

In summary, although HC-AIM is theoretically characterized by three interrelated functional components—AI awareness, AI monitoring, and AI-use regulation—the primary EFA supported a clear one-factor structure, with all nine items showing substantial loadings on a common factor. This conclusion was further supported by the complementary parallel analysis and the polychoric-correlation PAF, which also indicated a one-factor solution. The sensitivity analysis excluding invariant responders yielded a possible secondary factor under the reduced-sample condition, but the evidence for a substantively distinct second dimension was limited. Taken together, these findings suggest that the nine HC-AIMS items are more appropriately represented by a common underlying HC-AIM factor than by multiple empirically separable dimensions. This provided the basis for testing the hypothesized unidimensional structure in the confirmatory analyses of Study 2.

#### 2.4.3. Internal Consistency

The internal consistency of the HC-AIMS was further examined. The scale demonstrated good internal consistency, with a Cronbach’s α coefficient of 0.933.

Overall, Study 1 provided preliminary evidence supporting the predominantly unidimensional structure and good reliability of the HC-AIMS.

## 3. Study 2: Confirmatory Factor Analysis and Initial Validation of the HC-AIMS

### 3.1. Purpose of Study 2

Study 2 aimed to further examine the structural stability and construct validity of the Chinese version of the HC-AIMS. Specifically, an independent sample was used to conduct CFA to validate the predominantly unidimensional structure identified in Study 1. We also examined the relationships between HC-AIM and general metacognition. Because HC-AIM was conceptualized as a context-specific extension of traditional metacognition in AI-mediated cognitive environments, we expected it to be positively associated with general metacognition. The association between the two constructs was therefore examined to assess the convergent validity of the HC-AIMS.

### 3.2. Methods

#### 3.2.1. Participants

A total of 400 participants (Sample 2) were recruited using the same convenience-sampling procedure as described for Sample 1. Specifically, participants were recruited through general education courses in university classroom settings and completed the questionnaire online. As with Sample 1, no conventional response rate could be calculated because participation was voluntary and no fixed number of individuals was formally invited to participate.

Participants ranged in age from 18 to 30 years (M = 21.92, SD = 2.36), including 173 women and 227 men. All participants had prior experience using generative AI tools, such as ChatGPT, DeepSeek, or Kimi. The frequency of generative AI use was assessed using a five-point scale ranging from very frequent to very infrequent. All participants reported a frequency rating of 4 or higher, indicating that they had relatively frequent experience using generative AI tools. All participants voluntarily participated in the study and provided informed consent before completing the measures. No monetary or other compensation was provided. The study was approved by the Institutional Review Board of China University of Geosciences (Beijing) (No. 20251225). The recruitment, informed-consent, compensation, data-quality screening, and exclusion procedures were identical to those described for Sample 1. Specifically, responses were excluded if the questionnaire was incomplete, if completion time was shorter than one-third (<83 s) or longer than three times (>747 s) the mean completion time of 10 pilot respondents (M = 249 s), or if multiple responses originated from the same IP address (only the first submission would have been retained). No responses were excluded by any criterion (incomplete: 0; too short: 0; too long: 0; duplicate IP: 0), and all 400 responses were retained for analysis.

#### 3.2.2. Measures

##### Human-Centered AI Metacognition

HC-AIM was assessed using the Chinese version of the HC-AIMS developed in Study 1. The scale consists of nine items rated on a 5-point Likert scale. Higher scores indicate higher levels of HC-AIM.

##### General Metacognition

General metacognitive ability was assessed using the 19-item Metacognitive Awareness Inventory (MAI-19) revised by Harrison and Vallin (2018). Each item (e.g., “Before starting a task, I think about what I really need to learn or solve.”) was rated on a 5-point Likert scale. The scale assesses individuals’ ability to plan, monitor, and regulate their own cognitive processes. Higher total scores indicate higher levels of general metacognitive ability. In the present study, the scale demonstrated excellent internal consistency, with a Cronbach’s α coefficient of 0.962.

##### Instrumental AI Dependence

The Instrumental Dependency Subscale of the Large Language Model Dependency Questionnaire (LLM-D12) developed by Yankouskaya et al. (2025) was used to measure individuals’ instrumental dependence on AI tools. This subscale primarily assesses the extent to which individuals tend to over-rely on AI to complete cognitive tasks. The subscale consists of six items (e.g., “I sometimes use AI without realizing it and fail to notice how much time I have spent immersed in it” and “Even when I could make a decision through my own effort, I still turn to AI for decision support”) and adopts a 6-point Likert scoring format. Higher total scores indicate a stronger tendency to excessively rely on AI when performing cognitive tasks. The scale demonstrated good internal consistency in the present study (Cronbach’s α = 0.868).

#### 3.2.3. Data Analysis

Several analyses were conducted to examine the psychometric properties and construct validity of the Chinese version of the HC-AIMS. Internal consistency was assessed using Cronbach’s alpha (α). Pearson correlations were also computed to examine the associations of HC-AIM with general metacognition and instrumental AI dependence.

The dimensionality of the HC-AIMS was examined using confirmatory factor analysis (CFA) in Mplus 8.3. The nine HC-AIMS items were rated on a five-point Likert-type scale and were treated as approximately continuous indicators in the primary CFA, which used maximum likelihood (ML) estimation. Methodological research suggests that indicators with five or more response categories can often be treated as approximately continuous (Robitzsch, 2020; Rhemtulla et al., 2012; Huh & Gim, 2025; McNeish, 2024).

There were no missing responses in Study 2. Model fit was evaluated using χ^2^, χ^2^/df, CFI, TLI, RMSEA with its 90% confidence interval, and SRMR. The hypothesized one-factor model of the HC-AIMS was compared with theoretically motivated alternative factor structures. Correlated residuals were specified only when supported by theoretically interpretable overlap in item content.

To examine the construct distinguishability of HC-AIM from general metacognition, one-factor and correlated-factor CFA models were compared. Because the general metacognition measure comprises two dimensions, these two dimensions were retained in the correlated-factor model rather than being collapsed into a single first-order factor. Thus, the comparison model represented HC-AIM as one factor and general metacognition as a two-dimensional construct. Model fit and the latent correlation between HC-AIM and general metacognition were considered when interpreting their empirical distinguishability.

### 3.3. Results

#### 3.3.1. Confirmatory Factor Analysis

To further validate the one-factor structure identified in Study 1, a CFA was conducted to examine the model fit. A one-factor model was specified, with all nine items loading onto the latent construct of HC-AIM. The initial model showed an acceptable but not optimal fit to the data, χ^2^(27) = 132.09, χ^2^/df = 4.89, CFI = 0.940, TLI = 0.920, RMSEA = 0.099, and SRMR = 0.037. Although the CFI, TLI, and SRMR met commonly used criteria for acceptable fit, the RMSEA was close to 0.10, indicating some degree of model misfit. The composite reliability (CR) was 0.900, and the average variance extracted (AVE) was 0.504.

Inspection of the modification indices suggested three theoretically interpretable residual covariances: A4–A5, A6–A8, and A1–A2. These item pairs showed meaningful content overlap within the broader metacognitive framework. Specifically, A1 and A2 both concern individuals’ knowledge of AI capabilities and their appropriate application across contexts; A4 and A5 both involve monitoring AI-generated information and responding to inconsistencies; and A6 and A8 both concern the regulation of one’s reliance on AI according to one’s level of dependence and task demands. Accordingly, residual covariances were specified between these three pairs.

The revised one-factor model demonstrated substantially improved fit, χ^2^(24) = 70.39, χ^2^/df = 2.93, CFI = 0.974, TLI = 0.960, RMSEA = 0.070, 90% CI [0.051, 0.089], and SRMR = 0.027. The final model showed good composite reliability (CR = 0.897). The AVE was 0.494, which was marginally below the conventional 0.50 criterion but very close to the recommended threshold. Taken together, the CFA results provided further support for a predominantly unidimensional representation of the HC-AIMS in an independent sample.

Because the Study 1 sensitivity analysis yielded a two-factor solution in parallel analysis and PAF, we additionally tested a competing two-factor model based on the corresponding item pattern. Specifically, A1–A4 were specified to load on Factor 1, and A5–A9 were specified to load on Factor 2. The two-factor model showed acceptable but not excellent fit, χ^2^(26) = 118.103, χ^2^/df = 4.542, CFI = 0.948, TLI = 0.928, RMSEA = 0.094, and SRMR = 0.036. However, the correlation between the two latent factors was extremely high (r = 0.939, 95% CI [0.902, 0.976], *p* < .001), indicating limited empirical separation between the two factors. More importantly, the A1–A4 versus A5–A9 partition did not correspond to a coherent theoretical distinction within HC-AIM. Specifically, A1–A3 primarily concern AI awareness, whereas A4 concerns AI monitoring; conversely, A5 concerns AI monitoring, whereas A6–A9 primarily concern AI-use regulation. Thus, the two monitoring items were divided across the two factors, while conceptually different processes were combined within each factor. Taken together, although the two-factor model was statistically estimable and showed acceptable overall fit, the high latent correlation and the lack of a clear theoretical interpretation of the factor partition provided limited support for treating the two factors as substantively distinct dimensions.

As a sensitivity analysis (reported in the Supplementary Materials), the CFA was repeated after excluding participants who showed invariant response patterns across all nine HC-AIMS items, resulting in a subsample of 255 participants. The findings were consistent with the primary CFA, with the same one-factor structure and theoretically justified residual covariances providing good model fit, suggesting that the observed factor structure was robust to the exclusion of invariant response patterns.

##### Internal Consistency

The results showed that the Chinese version of the HC-AIMS demonstrated good internal consistency in Study 2, with a Cronbach’s α of 0.898.

##### Descriptive Statistics of the Main Variables

Descriptive statistics for the main variables in Study 2 are presented in Table 4. The skewness values ranged from −0.328 to 0.382, and the kurtosis values ranged from −0.628 to 0.174. All absolute values were below 1, indicating that the distributions of the three variables were approximately normal and suitable for parametric analyses.

##### Validity Assessment

Correlation analyses showed that HC-AIM was significantly positively correlated with general metacognitive ability, *r* = 0.700, *p* < 0.001, indicating that the HC-AIMS has good convergent validity.

Given the relatively strong association between HC-AIM and general metacognition observed in Study 2, we conducted an additional series of confirmatory factor analyses to examine whether the two constructs were empirically distinguishable. Specifically, we first compared a one-factor model, in which all HC-AIMS items (A1–A9) and general metacognition items (C1–C19) loaded on a single latent factor, with a two-factor model in which HC-AIM and general metacognition were represented as separate but correlated latent factors. The one-factor model showed poor fit to the data, χ^2^(350) = 2134.547, χ^2^/df = 6.10, CFI = 0.786, TLI = 0.769, RMSEA = 0.113, and SRMR = 0.073. In contrast, the two-factor model demonstrated substantially improved but still suboptimal fit, χ^2^(349) = 1493.115, χ^2^/df = 4.28, CFI = 0.863, TLI = 0.851, RMSEA = 0.091, and SRMR = 0.053. The correlation between the two latent factors was 0.735 (95% CI [0.683, 0.788], *p* < 0.001), indicating a strong association between HC-AIM and general metacognition. Although the two-factor model fit better than the one-factor model, its absolute fit did not provide strong evidence for a clear separation between the two constructs. Furthermore, both factors nevertheless showed acceptable composite reliability and average variance extracted (CR/AVE = 0.901/0.506 for HC-AIM and 0.963/0.578 for general metacognition).

Because general metacognition comprises two theoretically defined dimensions, we subsequently conducted an additional CFA that explicitly retained this multidimensional structure. In this model, HC-AIM was specified as a first-order factor indicated by A1–A9, whereas general metacognition was specified as a second-order factor indicated by two first-order dimensions (B1–B8 and B9–B19). The revised model showed improved, although still marginal, model fit, χ^2^(347) = 1272.559, χ^2^/df = 3.667, CFI = 0.889, TLI = 0.879, RMSEA = 0.082, and SRMR = 0.050. The correlation between HC-AIM and the higher-order general metacognition factor was 0.756 (95% CI [0.704, 0.809], *p* < 0.001). HC-AIM showed CR = 0.901 and AVE = 0.506, whereas the higher-order general metacognition factor showed CR = 0.945 and AVE = 0.895.

Taken together, the one-factor model fit substantially worse than the models distinguishing HC-AIM from general metacognition, and explicitly modeling the multidimensional structure of general metacognition further improved model fit. However, because the revised higher-order model showed only marginal overall fit and the latent correlation remained relatively high, these findings should not be interpreted as definitive evidence of discriminant validity. Rather, they provide preliminary evidence that HC-AIM is empirically distinguishable from, while substantially related to, general metacognition. This pattern is consistent with the theoretical conceptualization of HC-AIM as a context-specific metacognitive capability applied to AI-mediated cognitive environments rather than as a construct wholly independent of general metacognitive ability.

We also examined the association between HC-AIM and instrumental AI dependence as an exploratory external correlate. In the full Study 2 sample, HC-AIM was significantly negatively correlated with instrumental AI dependence, *r* = −0.133, *p* = .008. However, this association was not statistically significant in the sensitivity analysis after excluding respondents with invariant response patterns (*r* = −0.074, *p* = .236). Thus, the observed association between HC-AIM and instrumental AI dependence was sensitive to the treatment of invariant response patterns and was not consistently replicated across the two samples. Accordingly, instrumental AI dependence was not considered a robust external criterion for HC-AIM, and this association was not used as primary evidence of criterion-related validity.

## 4. Study 3: Examining the Construct Distinctiveness and Validity of HC-AIM

### 4.1. Purpose of Study 3

Study 3 was designed to further evaluate the reliability and construct validity of the Chinese version of the HC-AIMS and to clarify its nomological position in relation to theoretically relevant AI-related constructs. First, we sought to replicate the factor structure of the HC-AIMS in an independent sample and further examine its reliability. Second, we examined the relationships between HC-AIM and two closely related constructs, AI literacy and collaborative AI metacognition, and compared one-factor and two-factor measurement models to examine whether these constructs showed preliminary evidence of empirical distinguishability. Finally, we examined the association between HC-AIM and autonomous cognitive offloading as a self-reported criterion-related to AI-supported cognitive engagement, thereby extending the validation of HC-AIM beyond self-reported metacognitive and AI-related competencies to an AI-supported cognitive behavioral tendency.

### 4.2. Methods

#### 4.2.1. Participants

A total of 350 participants (Sample 3) were recruited using the same convenience-sampling procedure as described for Sample 1. Participants were recruited through general education courses in university classroom settings and completed the questionnaire online. As with Samples 1 and 2, no conventional response rate could be calculated because participation was voluntary and no fixed number of individuals was formally invited to participate.

Participants ranged in age from 16 to 22 years (M = 18.16, SD = 0.51), including 133 women and 217 men. Ten participants were younger than 18 years of age at the time of participation. The sample was recruited from university general education courses and included a small number of first-year undergraduate students who had not yet reached the age of 18. The study was approved by the Institutional Review Board of China University of Geosciences (Beijing) (No. 20251225). The approved IRB protocol explicitly covered the participation of minors. For these ten participants under the age of 18, verbal consent was first obtained from their parents or legal guardians by telephone. The minor participants then provided written informed consent themselves before completing the questionnaire. Adult participants provided written informed consent prior to participation. All participants voluntarily participated in the study, and no monetary or other compensation was provided.

All participants had prior experience using generative AI tools, such as ChatGPT, DeepSeek, or Kimi. The frequency of generative AI use was assessed using a five-point scale ranging from very frequent to very infrequent. All participants reported a frequency rating of 4 or higher, indicating that they had relatively frequent experience using generative AI tools. The recruitment, informed-consent, compensation, data-quality screening, and exclusion procedures were identical to those described for Sample 1. Specifically, responses were excluded if the questionnaire was incomplete, if completion time was less than one-third (<88 s) or more than three times (>792 s) the mean completion time of 10 pilot respondents (M = 264 s), or if multiple responses originated from the same IP address (only the first submission would have been retained). No responses were excluded by any criterion (incomplete: 0; too short: 0; too long: 0; duplicate IP: 0), and all 350 responses were retained for analysis.

#### 4.2.2. Measures

##### Human-Centered AI Metacognition

HC-AIM was assessed using the Chinese version of the HC-AIMS developed in Study 1. The scale consists of nine items rated on a 5-point Likert scale. Higher scores indicate higher levels of HC-AIM.

##### AI Literacy

AI literacy was assessed using the 10-item short version of the Meta AI Literacy Scale (MAILS) developed by Koch et al. (2024). Each item (e.g., “I can use artificial intelligence meaningfully to achieve my everyday goals.”) was rated on an 11-point self-report scale ranging from 0 (no ability at all) to 10 (very good ability). The scale assesses individuals’ competencies relevant to understanding, evaluating, and appropriately using AI. Higher total scores indicate greater AI literacy. In the present study, the scale demonstrated good internal consistency, with a Cronbach’s α coefficient of 0.901.

##### Collaborative AI Metacognition

Collaborative AI metacognition was assessed using the 11-item Collaborative AI Metacognition Scale developed by Sidra and Mason (2026). The scale was designed to capture metacognitive knowledge and processes involved in working with AI in collaborative human–AI contexts. Each item (e.g., “When working with the AI, I check task goals every now and then to make sure we’re on track.”) was rated on a 5-point Likert scale. Higher total scores indicate stronger collaborative AI metacognition. In the present study, the scale demonstrated good internal consistency, with a Cronbach’s α coefficient of 0.948.

##### Autonomous Cognitive Offloading

Autonomous cognitive offloading was assessed using the four-item self-report measure developed by Zhu et al. (2026). The scale captures the extent to which individuals use generative AI as a cognitive scaffold while retaining ownership and control over their own thinking. Zhu et al. (2026) conceptualized autonomous offloading as a distinct mode of cognitive engagement with generative AI in which users retain cognitive agency while benefiting from external cognitive support. The four items (e.g., “AI helps me think more broadly, but I make the final decisions myself”) were rated on a five-point Likert scale, with higher scores indicating greater use of AI in an autonomous, cognitively agentic manner. In the present study, the Cronbach’s α coefficient of the subscale was 0.749.

#### 4.2.3. Data Analysis

The confirmatory factor analyses in Study 3 followed the same estimation and model-evaluation procedures described for Study 2. Specifically, the five-point HC-AIMS items were treated as approximately continuous indicators and estimated using maximum likelihood (ML) in Mplus 8.3. No missing responses were present. Model fit was evaluated using χ^2^, χ^2^/df, CFI, TLI, RMSEA with its 90% confidence interval, and SRMR.

In addition to evaluating the one-factor structure of the HC-AIMS, we examined its construct distinguishability from AI literacy and Collaborative AI Metacognition. Because both comparison constructs are theoretically multidimensional, their established first-order dimensions were retained in the CFA models rather than collapsing all items into single first-order factors.

### 4.3. Results

#### 4.3.1. Confirmatory Factor Analysis

To further examine the factorial structure of the Chinese version of the HC-AIMS, a one-factor CFA was conducted in Study 3. The initial one-factor model showed an acceptable but not optimal fit to the data, χ^2^(27) = 123.504, χ^2^/df = 4.574, CFI = 0.930, TLI = 0.907, RMSEA = 0.101, and SRMR = 0.044. The CFI, TLI, and SRMR were within or close to conventional acceptable ranges, whereas the RMSEA indicated a relatively marginal fit. The HC-AIMS demonstrated satisfactory composite reliability (CR = 0.890), although the AVE was slightly below the conventional 0.50 criterion (AVE = 0.475).

Inspection of the modification indices indicated a substantial localized source of misfit involving the residual covariance between Items 1 and 2 (MI = 38.678). Given the conceptual similarity between the two items, their residual covariance was freely estimated. The modified one-factor model showed improved fit, χ^2^(26) = 86.717, χ^2^/df = 3.335, CFI = 0.956, TLI = 0.939, RMSEA = 0.082, 90% CI [0.063, 0.101], and SRMR = 0.038. The modified model also demonstrated good composite reliability (CR = 0.888), although the AVE remained slightly below 0.50 (AVE = 0.470). Overall, these results provided further support for a predominantly unidimensional representation of the HC-AIMS in Study 3.

#### 4.3.2. Internal Consistency

The results showed that the HC-AIMS demonstrated good internal consistency in Study 3, with a Cronbach’s α of 0.885.

#### 4.3.3. Relationships Between HC-AIM and AI-Related Constructs

To examine the nomological position of HC-AIM in relation to theoretically relevant AI-related competencies, its associations with AI literacy and collaborative AI metacognition were examined. HC-AIM was significantly and positively correlated with AI literacy, as assessed by the Meta AI Literacy Scale (MAILS), r = 0.644, *p* < 0.001. HC-AIM was also significantly and positively correlated with collaborative AI metacognition, r = 0.701, *p* < 0.001. These findings indicate that individuals with higher levels of HC-AIM also tended to report higher levels of AI literacy and collaborative AI metacognition.

To further examine whether these substantial associations reflected a common underlying construct, item-level CFA comparisons were conducted for HC-AIM and each related construct. For HC-AIM and AI literacy, a one-factor model in which all 19 items (9 for HC-AIM, 10 for AI literacy) were loaded on a single latent factor showed poor fit, χ^2^(152) = 1590.218, χ^2^/df = 10.462, CFI = 0.664, TLI = 0.622, RMSEA = 0.164, and SRMR = 0.099. A two-factor model specifying HC-AIM and AI literacy as separate but correlated latent factors showed improved but inadequate fit, χ^2^(151) = 1279.872, χ^2^/df = 8.476, CFI = 0.736, TLI = 0.701, RMSEA = 0.146, and SRMR = 0.083. The correlation between the two latent factors was 0.776 (95% CI [0.723, 0.830], *p* < 0.001). Although the two-factor model fit better than the one-factor model, its absolute fit remained poor. Because AI literacy comprises four theoretically defined dimensions, we additionally examined a model that explicitly retained this multidimensional structure. In this model, HC-AIM was specified as a first-order factor indicated by A1–A9, whereas AI literacy was specified as a second-order factor indicated by four first-order dimensions (B1–B4, B5–B6, B7–B8, and B9–B10). This revised two-construct model showed substantially improved fit, χ^2^(147) = 451.238, χ^2^/df = 3.070, CFI = 0.929, TLI = 0.917, RMSEA = 0.077 (90% CI [0.069, 0.085]), and SRMR = 0.067. The correlation between HC-AIM and the higher-order AI literacy factor was 0.779 (95% CI [0.715, 0.843], *p* < 0.001). HC-AIM showed CR = 0.889 and AVE = 0.474, whereas the higher-order AI literacy factor showed CR = 0.860 and AVE = 0.612. Thus, explicitly modeling the multidimensional structure of AI literacy substantially improved the representation of the data. Nevertheless, the relatively high latent correlation indicates that HC-AIM and AI literacy remain substantially related constructs.

A similar comparison was conducted for HC-AIM and collaborative AI metacognition. The one-factor model, in which all 20 items (9 for HC-AIM, 11 for collaborative AI metacognition) were loaded on a single latent factor, showed poor fit, χ^2^(170) = 1039.585, χ^2^/df = 6.115, CFI = 0.817, TLI = 0.795, RMSEA = 0.121, and SRMR = 0.071. In contrast, the two-factor model specifying HC-AIM and collaborative AI metacognition as separate but correlated latent factors showed substantially improved but less than ideal fit, χ^2^(169) = 624.257, χ^2^/df = 3.694, CFI = 0.904, TLI = 0.892, RMSEA = 0.088, and SRMR = 0.041. The correlation between the two latent factors was 0.758 (95% CI [0.704, 0.812], *p* < 0.001). Because collaborative AI metacognition comprises three theoretically defined dimensions, we additionally modeled its multidimensional structure. Specifically, HC-AIM was specified as a first-order factor indicated by A1–A9, whereas collaborative AI metacognition was specified as a second-order factor indicated by three first-order dimensions (C1–C4, C5–C8, and C9–C11). The revised model showed good overall fit, χ^2^(166) = 430.034, χ^2^/df = 2.590, CFI = 0.944, TLI = 0.936, RMSEA = 0.067 (90% CI [0.060, 0.075]), and SRMR = 0.036. The correlation between HC-AIM and the higher-order collaborative AI metacognition factor was 0.773 (95% CI [0.718, 0.827], *p* < 0.001). HC-AIM showed CR = 0.890 and AVE = 0.476, whereas the higher-order collaborative AI metacognition factor showed CR = 0.949 and AVE = 0.862. These results indicate that explicitly modeling the multidimensional structure of collaborative AI metacognition substantially improved the fit of the comparison model. However, the relatively high latent correlation suggests that the constructs still overlap substantially.

Taken together, the findings from Study 3 provide preliminary evidence of empirical distinguishability between HC-AIM and both AI literacy and collaborative AI metacognition when the multidimensional structures of the comparison measures are appropriately modeled. At the same time, the relatively high latent correlations between HC-AIM and AI literacy (0.779) and between HC-AIM and collaborative AI metacognition (0.773) indicate substantial conceptual and empirical relatedness. Thus, these findings should not be interpreted as definitive evidence of discriminant validity. Rather, they are consistent with the conceptualization of HC-AIM as a related but distinguishable, context-specific form of metacognitive regulation focused on recognizing, monitoring, and regulating AI involvement in human cognition.

#### 4.3.4. HC-AIM and Autonomous Cognitive Offloading

To further examine the criterion-related validity of the HC-AIMS, we examined its association with autonomous cognitive offloading. HC-AIM was significantly and positively correlated with autonomous cognitive offloading, *r* = 0.380, *p* < 0.001. This finding indicates that higher levels of HC-AIM were associated with greater self-reported engagement in autonomous cognitive offloading, providing additional self-reported criterion-related evidence for the validity of the HC-AIMS.

#### 4.3.5. Sensitivity Analysis

As a sensitivity analysis, we repeated the primary analyses for Study 3 after excluding 89 participants who selected the same response category across all nine HC-AIMS items, resulting in a reduced sample of N = 261. The HC-AIMS retained acceptable internal consistency (α = 0.810), and the one-factor CFA showed an overall acceptable fit after accounting for a theoretically interpretable residual covariance between A1 and A2, χ^2^(26) = 57.621, CFI = 0.946, TLI = 0.925, RMSEA = 0.068, and SRMR = 0.045. HC-AIM remained significantly and positively correlated with AI literacy (r = 0.655, *p* < 0.001), collaborative AI metacognition (r = 0.678, *p* < 0.001), and autonomous cognitive offloading (r = 0.209, *p* = 0.001). CFA comparisons with AI literacy and collaborative AI metacognition also yielded patterns consistent with the primary analyses, with models distinguishing HC-AIM from the respective comparison constructs showing better fit than corresponding one-factor models. Detailed sensitivity analyses are reported in the Supplementary Materials.

## 5. Discussion

### 5.1. Overview of the Present Study

The present research developed and provided an initial psychometric evaluation of the Chinese version of the HC-AIMS across three studies. Studies 1 and 2 supported its predominantly unidimensional structure and demonstrated good internal consistency, which were further replicated in Study 3. The findings also showed that HC-AIM was positively associated with general metacognition, AI literacy, and collaborative AI metacognition. Although these constructs were substantially related to HC-AIM, comparisons of theoretically specified factor models provided preliminary evidence that HC-AIM and each related construct are better represented as distinct but correlated factors than as a single common factor. Given the substantial latent associations, these findings are interpreted as preliminary evidence of empirical distinguishability rather than definitive evidence of discriminant validity.

Study 3 further showed that HC-AIM was positively associated with autonomous cognitive offloading, providing additional self-reported criterion-related evidence for the validity of the Chinese version of the HC-AIMS. Taken together, these findings provide initial evidence that HC-AIM is a contextualized, human-centered form of metacognitive regulation that is closely related to broader metacognitive and AI-related competencies while emphasizing the regulation of AI participation in human cognition. The present findings provide a basis for further validation of the HC-AIMS using more diverse samples, behavioral or performance-based indicators, and tests of incremental validity.

### 5.2. Human-Centered AI Metacognition as a Contextualized Extension of Metacognition

Across the three studies, the findings provide converging but preliminary evidence for understanding HC-AIM as a contextualized form of metacognitive regulation rather than as an entirely independent psychological capacity. In Study 2, HC-AIM was strongly positively associated with general metacognition, indicating substantial continuity between the regulation of cognition in general and the regulation of cognition in AI-mediated contexts. This finding is consistent with classic accounts of metacognition, which emphasize individuals’ awareness, monitoring, and regulation of their own cognitive processes (Flavell, 1979; Nelson & Narens, 1990; Schraw & Dennison, 1994). At the same time, the relatively strong association between the two constructs raises an important question concerning whether HC-AIM represents merely a domain-specific expression of general metacognitive ability or captures additional regulatory demands associated with AI-mediated cognition.

The CFA comparisons provide preliminary evidence that these constructs are related but not reducible to a single underlying dimension. Using the item-level data from Study 2, the two-factor model distinguishing HC-AIM from general metacognition showed substantially better fit than the corresponding one-factor model, although the absolute fit of the two-factor model remained less than ideal. In addition, when the multidimensional structure of general metacognition was explicitly modeled using a higher-order factor, model fit improved further, but the overall fit remained marginal and the latent correlation between HC-AIM and general metacognition remained substantial. This pattern suggests that, despite their substantial association, the items of HC-AIM and general metacognition are better represented as reflecting two related constructs rather than a single undifferentiated metacognitive dimension. Theoretically, this partial differentiation can be understood in terms of the regulatory target. General metacognition primarily concerns monitoring and regulating one’s own cognitive activities, whereas HC-AIM applies metacognitive regulation to situations in which an AI system can actively participate in those activities. Individuals must therefore not only monitor their own cognition but also recognize AI capabilities and limitations, evaluate AI-generated information, and regulate the extent and manner of AI participation. From this perspective, HC-AIM may involve a contextualized extension of established metacognitive processes rather than a qualitatively independent cognitive capacity.

This interpretation is consistent with the broader perspective of extended cognition, which emphasizes that cognitive processes can involve interactions between individuals and external resources (Clark & Chalmers, 1998). The involvement of AI does not necessarily constitute an entirely new cognitive system; rather, it creates a cognitive environment in which established metacognitive processes must operate in relation to an external intelligent system. HC-AIM may therefore be understood as a contextualized extension of metacognition that incorporates AI-specific regulatory demands while retaining the broader metacognitive foundation of awareness, monitoring, and regulation. Importantly, the present findings do not support treating HC-AIM and general metacognition as interchangeable, but neither do they establish them as fully independent constructs. Instead, they suggest that HC-AIM occupies a specific position within the broader metacognitive system: it represents the application of metacognitive regulation to the management of AI participation in human cognition. Accordingly, the theoretical contribution of HC-AIM should be understood as specifying a context-specific regulatory focus within the broader metacognitive system, rather than establishing a clearly independent psychological capacity.

### 5.3. HC-AIM and AI-Related Competencies: Related but Preliminary Evidence of Empirical Distinguishability

Study 3 further clarified the conceptual position of HC-AIM in relation to AI-related competencies. HC-AIM was positively associated with AI literacy and collaborative AI metacognition, indicating that HC-AIM shares substantial variance with individuals’ knowledge and understanding of AI as well as their metacognitive regulation of human–AI collaboration. These associations are theoretically expected. Individuals who understand AI capabilities and limitations and who are able to reflect on cognitive processes involving AI may also be better positioned to regulate AI’s participation in their own cognition. Thus, the present findings do not support conceptualizing HC-AIM as unrelated to existing AI-related competencies. Rather, they place HC-AIM within a broader network of closely related constructs concerned with human cognition in AI-mediated environments.

At the same time, the CFA comparisons provide preliminary evidence that substantial associations do not necessarily imply construct equivalence. For both AI literacy and collaborative AI metacognition, models that represented HC-AIM and the corresponding comparison construct as separate but correlated factors fit substantially better than models that combined their items into a single factor. Moreover, when the multidimensional structures of AI literacy and collaborative AI metacognition were explicitly modeled using higher-order factors, model fit improved considerably, with both revised models showing acceptable overall fit. Nevertheless, the latent correlations between HC-AIM and the higher-order AI-related constructs remained relatively high. Accordingly, these findings are better interpreted as evidence of preliminary empirical distinguishability rather than definitive evidence of discriminant validity.

This distinction is particularly important for understanding the specific conceptual positioning of HC-AIM. AI literacy primarily concerns individuals’ knowledge and understanding of AI, whereas collaborative AI metacognition focuses on metacognitive processes involved in coordinating cognitive activities with AI. HC-AIM, in contrast, places particular emphasis on regulating the role and extent of AI participation in one’s own cognition while preserving cognitive autonomy. These constructs therefore differ in their primary regulatory or psychological focus, even though they are expected to overlap substantially in individuals’ actual experiences of AI-mediated cognition.

Accordingly, the contribution of HC-AIM should not be framed as identifying a completely separate cognitive ability that operates independently of general metacognition or existing AI-related competencies. Rather, HC-AIM represents a specific regulatory focus within this broader conceptual network. Its central concern is the individual’s metacognitive management of the human–AI cognitive relationship: recognizing when AI participation is appropriate, monitoring AI-generated information, and regulating AI involvement in ways that allow the individual to remain actively engaged in cognitive activities. This formulation also helps explain why HC-AIM can be substantially correlated with general metacognition, AI literacy, and collaborative AI metacognition while nonetheless showing preliminary evidence of empirical distinguishability from these constructs. More broadly, the findings position HC-AIM as a context-specific manifestation of metacognitive regulation that becomes particularly relevant when cognition is distributed across human and AI agents.

### 5.4. HC-AIM and Autonomous Cognitive Offloading

The positive association between HC-AIM and autonomous cognitive offloading in Study 3 provides self-reported criterion-related evidence that complements the psychometric findings of the present research. Whereas the other validity analyses primarily examined HC-AIM in relation to conceptually related psychological constructs, its association with autonomous cognitive offloading indicates that individual differences in metacognitive regulation of AI involvement are also reflected in how people report incorporating AI into their cognitive activities. In particular, the finding suggests that higher HC-AIM is associated with a greater tendency to report using AI as an external cognitive resource without necessarily displacing one’s own cognitive engagement. This is theoretically important because it indicates that human-centered AI use should not be understood simply in terms of reducing AI reliance or minimizing external cognitive support. Rather, the relevant distinction concerns whether individuals remain capable of regulating their engagement with AI while incorporating its cognitive contributions.

This finding also reinforces the regulatory nature of HC-AIM. The construct is not defined by a particular level of AI use or by avoidance of cognitive offloading; instead, it concerns the capacity to manage AI involvement in accordance with the demands of one’s cognitive activities. The positive relationship with autonomous cognitive offloading is consistent with this view, suggesting that effective metacognitive regulation may coexist with, and potentially facilitate, productive forms of AI-supported cognition. At the same time, because the evidence is correlational, the present findings do not establish that HC-AIM leads to autonomous cognitive offloading. Future longitudinal and experimental research could examine whether stronger metacognitive regulation of AI involvement contributes to more autonomous patterns of AI-supported cognition over time. Together, the findings provide complementary self-reported criterion-related evidence for the HC-AIMS and further support its conceptualization as a human-centered form of metacognitive regulation that concerns not only how individuals evaluate AI but also how they incorporate AI into their ongoing cognitive activities.

### 5.5. Implications for Understanding Intelligence in the AI Era

The present findings also have broader implications for understanding intelligent functioning in AI-mediated environments. Traditional research on human intelligence has primarily focused on individuals’ abilities to reason, learn, and solve problems, typically conceptualizing cognition as occurring primarily within the individual (Colom et al., 2010; Neisser et al., 1996; Sternberg, 2012). As AI becomes increasingly integrated into cognitive activities, however, individuals can draw on external intelligent resources when performing tasks that were previously undertaken primarily through their own cognitive processes. This development raises a broader question concerning how individuals regulate the relationship between their own cognition and external intelligent resources.

The present findings suggest that metacognitive regulation of AI involvement may be relevant to this emerging form of intelligent functioning. The positive association between HC-AIM and general metacognition indicates that regulating AI participation is grounded in broader cognitive-regulatory processes, while the associations with AI-related competencies and autonomous cognitive offloading suggest that this regulatory capacity is relevant to how individuals incorporate AI into their cognitive activities. From this perspective, intelligent functioning in AI-mediated environments may involve not only what individuals can accomplish with the assistance of AI, but also how they monitor and regulate the role of AI while remaining actively engaged in their own cognitive processes.

This perspective does not imply that AI should be excluded from human cognitive activities. Rather, it highlights the importance of understanding how human and artificial cognitive resources can be coordinated while preserving individuals’ active role in cognition. Future research on intelligence in the AI era may therefore benefit from considering not only cognitive performance with intelligent systems, but also the metacognitive processes through which individuals regulate their participation in AI-supported cognition.

### 5.6. Practical Implications

The findings have several practical implications for AI education and the design of AI-supported learning and work environments.

First, AI literacy education and professional training could extend beyond technical knowledge and operational skills to include metacognitive regulation of AI involvement. Training programs could provide opportunities for learners to identify the capabilities and limitations of AI systems, critically evaluate AI-generated information, and consider when and how AI assistance is appropriate for a particular cognitive task. Such activities may help individuals develop a more deliberate approach to incorporating AI into their own cognitive processes rather than simply focusing on obtaining efficient AI outputs.

Second, AI systems could be designed to support users’ metacognitive engagement with AI-generated information. For example, systems could communicate uncertainty or limitations, encourage users to evaluate important outputs independently, and provide prompts for reflection when tasks require substantial human judgment. Such features may support active human engagement with AI-assisted cognition rather than simply optimizing the convenience or efficiency of AI interaction.

### 5.7. Limitations and Future Directions

First, a limitation concerns the representativeness of the samples. All studies relied on convenience sampling of university students recruited through general education courses, and all participants had prior experience using generative AI. The samples were therefore relatively young and educationally homogeneous, and they may not adequately represent individuals from other age groups, educational backgrounds, or levels of generative AI experience. Future research should replicate the findings in more diverse populations, including individuals from different age groups, educational and occupational backgrounds, and levels of generative AI experience, to further examine the generalizability of the HC-AIMS.

Second, the present study relied primarily on self-report measures. Although self-report is appropriate for assessing perceived cognitive tendencies, it cannot establish whether HC-AIM is reflected in actual cognitive behavior. Future research should therefore incorporate directly observed behavioral measures and performance-based indicators, such as AI-use logs, patterns of prompt revision, independent verification behavior, decisions to accept or reject AI recommendations, and performance on tasks requiring critical evaluation of AI-generated information. Such measures would provide stronger evidence of how HC-AIM operates in real-world AI use and would help determine whether the self-reported tendencies examined in the present study correspond to observable patterns of AI-supported cognition.

Third, another limitation concerns the relatively high proportion of invariant responding on the HC-AIMS. In Study 1, 79 of 210 participants (37.6%) selected the same response category across all nine items, with comparable proportions in Study 2 (145/400, 36.3%) and Study 3 (89/350, 25.4%). Invariant responding does not necessarily indicate invalid data, but it may reflect response style, ceiling tendencies, or the high semantic interrelatedness of the items—the latter being consistent with the deliberate conceptualization of HC-AIM as a unified regulatory capability. The present data cannot distinguish between these possibilities. Analytically, sensitivity analyses excluding invariant responders were conducted in all three studies, and the main findings were largely replicated in Studies 2 and 3; however, the unidimensional conclusion in Study 1 was sensitive to the exclusion of invariant responders. Future research should therefore incorporate response-style indicators, such as person-fit statistics and intra-individual response variability, and test measurement invariance across respondents with differing response tendencies.

Fourth, the cross-sectional design limits causal interpretations of the relationship between HC-AIM and autonomous cognitive offloading in Study 3. Although the two constructs were positively correlated, this association does not establish a causal relationship. Future longitudinal or experimental research could further examine this relationship.

Fifth, future research should examine the incremental validity of HC-AIM over and above general metacognition, AI literacy, and collaborative AI metacognition. Because HC-AIM was substantially correlated with these constructs in the present studies, it remains to be established whether HC-AIM explains meaningful variance in relevant outcomes—such as autonomous cognitive offloading and critical evaluation of AI-generated information—that is not already captured by these established measures. Demonstrating such incremental validity, ideally in combination with behavioral and performance-based indicators, would provide stronger evidence for the construct’s distinctive explanatory value.

Finally, another limitation concerns the cross-linguistic validation of the HC-AIMS. The present research established the psychometric properties of the Chinese version, which was the version used for item development, expert review, data collection, and validation. Although the English translation was prepared by the author team and subsequently reviewed and revised by two professors of English, it was not used for data collection and was not subjected to a formal translation/back-translation procedure or independent psychometric validation. Therefore, the psychometric properties established in the present studies should not be assumed to generalize directly to English-speaking populations. Future research should evaluate the English translation in appropriate English-speaking samples and examine its reliability, factor structure, and validity, as well as its measurement equivalence across language groups where appropriate.

## 6. Conclusions

The present study developed the Chinese version of the HC-AIMS and provided an initial psychometric evaluation across three studies. The findings generally supported a predominantly unidimensional representation of the scale and indicated good internal consistency across multiple samples. HC-AIM was positively associated with general metacognition, AI literacy, collaborative AI metacognition, and autonomous cognitive offloading. Comparisons with theoretically related constructs further provided preliminary evidence of empirical distinguishability, although the substantial associations between these constructs indicate that HC-AIM is closely embedded within a broader network of metacognitive and AI-related competencies.

Overall, HC-AIM can be understood as a contextualized form of metacognitive regulation that focuses on how individuals regulate AI involvement in their cognitive activities while maintaining cognitive autonomy. HC-AIM is therefore best understood as a context-specific regulatory focus rather than as a clearly independent psychological capacity. The Chinese version of the HC-AIMS provides an initial, theoretically grounded measure for future research on metacognition and cognitive autonomy in AI-mediated environments. Further validation using more diverse samples, behavioral and performance-based indicators, and incremental-validity designs is needed to clarify the construct’s broader psychometric properties and its potential incremental contribution beyond existing metacognitive and AI-related measures.

## Figures and Tables

**Table 1:** Standardized Factor Loadings of the HC-AIMS in Study 1 (Pearson Correlation Matrix, *N* = 210).

Item	Factor Loading
A4 When using AI-generated answers, I check in real time whether the reasoning is logically sound.	0.865
A2 I know which learning tasks are suitable for AI assistance and which require me to work independently.	0.806
A9 After using AI, I reflect on the experience to improve how I use AI in the future (e.g., keeping track of effective prompt templates).	0.798
A7 When AI does not provide useful support, I reconsider how much I should rely on AI and adjust its role in the task accordingly.	0.788
A1 I clearly understand the strengths and limitations of AI tools (e.g., their potential to generate incorrect information).	0.786
A8 I adjust my level of reliance on AI according to task difficulty (e.g., trying to complete simple tasks independently whenever possible).	0.761
A6 I can recognize whether I am becoming overly dependent on AI (e.g., copying answers directly without thinking for myself).	0.755
A3 I consider the strengths and limitations of different AI tools when deciding how they should be used for a cognitive task.	0.746
A5 When an AI tool provides conflicting information, I pause and actively verify it (e.g., by consulting textbooks or authoritative sources).	0.728

*Note.* Principal axis factoring based on the Pearson correlation matrix. One factor was extracted. The one-factor solution explained 61.226% of the total variance after extraction. Analyses were based on the Chinese version of the HC-AIMS.

**Table 2:** Parallel Analysis Results for the HC-AIMS in Study 1 (*N* = 210).

Factor	Actual Eigenvalue	Random Simulated Mean	Resampled Simulated Mean
1	6.265	0.544	0.641
2	0.273	0.241	0.293
3	0.118	0.161	0.194
4	0.009	0.096	0.111
5	−0.009	0.029	0.032
6	−0.054	−0.032	−0.041
7	−0.093	−0.095	−0.112
8	−0.111	−0.157	−0.188
9	−0.133	−0.243	−0.289

*Note.* Parallel analysis was based on polychoric correlations (100 simulations). Analyses were based on the Chinese version of the HC-AIMS.

**Table 3:** Standardized Factor Loadings of the HC-AIMS in Study 1 (Polychoric Correlation Matrix, *N* = 210).

Item	Factor Loading
A4 When using AI-generated answers, I check in real time whether the reasoning is logically sound.	0.911
A2 I know which learning tasks are suitable for AI assistance and which require me to work independently.	0.854
A9 After using AI, I reflect on the experience to improve how I use AI in the future (e.g., keeping track of effective prompt templates).	0.850
A7 When AI does not provide useful support, I reconsider how much I should rely on AI and adjust its role in the task accordingly.	0.846
A1 I clearly understand the strengths and limitations of AI tools (e.g., their potential to generate incorrect information).	0.840
A8 I adjust my level of reliance on AI according to task difficulty (e.g., trying to complete simple tasks independently whenever possible).	0.815
A6 I can recognize whether I am becoming overly dependent on AI (e.g., copying answers directly without thinking for myself).	0.810
A3 I consider the strengths and limitations of different AI tools when deciding how they should be used for a cognitive task.	0.796
A5 When an AI tool provides conflicting information, I pause and actively verify it (e.g., by consulting textbooks or authoritative sources).	0.781

*Note.* Extraction method: principal axis factoring based on polychoric correlations. Analyses were based on the Chinese version of the HC-AIMS.

**Table 4:** Descriptive statistics and correlation analysis for main variables.

	*M* ± *SD*	*Skewness*	*Kurtosis*	1	2	3
1. HC-AIM	34.75 ± 5.54	0.047	−0.628	—		
2. GM	70.54 ± 11.42	0.382	−0.305	0.700 ***	—	
3. IAD	21.44 ± 5.62	−0.328	0.174	−0.133 **	−0.202 ***	—

*Note.* 1 = HC-AIM, 2 = GM, 3 = IAD. ** *p* < 0.01, *** *p* < 0.001 (two-tailed).

## Data Availability

Data is available as Supplementary Material.

## Supplementary Materials

The following supporting information can be downloaded at: https://www.mdpi.com/article/10.3390/jintelligence14100270/s1, The results of the sensitivity analysis and the items of HC-AIMS can be downloaded in Supplementary Materials.
