# Supplementary material for: Human-Centered AI Metacognition: Development and Validation of a Scale for Preserving Cognitive Autonomy in Human–AI Collaboration
Source: J Intell. 2026 Oct 20;14(10):270. doi: 10.3390/jintelligence14100270 (PMC13706084; doi:10.3390/jintelligence14100270)
Supplement: Supplementary file 1 [file jintelligence-14-00270-s001.zip › final_ sensitivity analysis.pdf]

Sensitivity Analysis of Study 1

To examine whether the one-factor solution identified in Study 1 was driven by respondents exhibiting invariant response patterns, we conducted a sensitivity analysis after excluding participants who selected the same response category across all nine HC-AIMS items. This procedure resulted in a reduced sample of $N = 131$.

EFA was conducted to examine the underlying factor structure of the HC-AIMS. A polychoric correlation matrix was used as the basis for factor analysis. The number of factors to retain was determined using both parallel analysis (PA) and principal axis factoring (PAF) based on the polychoric correlation matrix.

The reduced sample remained suitable for factor analysis. The Kaiser–Meyer–Olkin (KMO) measure of sampling adequacy was 0.88, and Bartlett's test of sphericity was significant, $\chi^2(36) = 385.71$, $p < .001$. The polychoric correlations remained uniformly positive, although they were generally lower than those observed in the full sample.

Parallel analysis yielded a somewhat different result in the reduced sample. The first observed eigenvalue was 4.268, substantially exceeding the mean eigenvalue obtained from random data (0.647) and the resampled eigenvalue (0.786). The second observed eigenvalue was 0.499, which was slightly higher than the corresponding random-data (0.318) and resampled (0.37) eigenvalues. Thus, parallel analysis

statistically indicated a two-factor solution in the reduced sample. The results of the parallel analysis were presented in Table S1.

Table S1 Parallel Analysis Results for the HC-AIMS in Study 1 *(N = 131)*

Factor	Actual Eigenvalue	Random Simulated Mean	Resampled Simulated Mean
1	4.268	0.647	0.786
2	0.499	0.318	0.37
3	0.182	0.205	0.25
4	0.028	0.116	0.148
5	−0.013	0.035	0.04
6	−0.084	−0.047	−0.049
7	−0.184	−0.120	−0.151
8	−0.199	−0.198	−0.246
9	−0.229	−0.309	−0.362

To further examine the factor structure suggested by the parallel analysis, PAF was conducted. The resulting pattern matrix showed a statistically recognizable two-factor structure, with A1–A4 loading primarily on one factor and A5–A9 loading primarily on the other. The detailed factor loadings were presented in Table S2. The two extracted factors together accounted for 55.01% of the total variance. Although the PAF produced a statistically recognizable two-factor pattern, the resulting item grouping did not correspond closely to the theoretically defined content domains of HC-AIM. Specifically, A1–A3 primarily assess awareness of AI capabilities, limitations, and task suitability, whereas A4 assesses monitoring of AI-generated information. In contrast, A5 also concerns AI monitoring, whereas A6–A9 primarily concern regulation of AI reliance and AI involvement. Thus, the two-factor pattern separates

the two conceptually related monitoring items (A4 and A5) across different factors, while combining different theoretical processes within each factor.

Table S2 Standardized Factor Loadings of the HC-AIMS in Study 1 (N = 131)

Item	Factor 1	Factor 2
A2 I know which learning tasks are suitable for AI assistance and which require me to work independently.	**0.888**	
A3 I consider the strengths and limitations of different AI tools when deciding how they should be used for a cognitive task.	**0.740**	
A4 When using AI-generated answers, I check in real time whether the reasoning is logically sound.	**0.727**	
A1 I clearly understand the strengths and limitations of AI tools (e.g., their potential to generate incorrect information).	**0.595**	
A8 I adjust my level of reliance on AI according to task difficulty (e.g., trying to complete simple tasks independently whenever possible).		**0.803**
A6 I can recognize whether I am becoming overly dependent on AI (e.g., copying answers directly without thinking for myself).		**0.671**
A7 When AI does not provide useful support, I reconsider how much I should rely on AI and adjust its role in the task accordingly.		**0.669**
A9 After using AI, I reflect on the experience to improve how I use AI in the future (e.g., keeping track of effective prompt templates).		**0.591**
A5 When an AI tool provides conflicting information, I pause and actively verify it (e.g., by consulting textbooks or authoritative sources).		**0.412**

Note. Extraction method: Principal Axis Factoring (PAF) based on the polychoric correlation matrix.

Overall, the sensitivity analysis indicated that exclusion of participants with invariant response patterns resulted in a statistical indication of two factors. However, the resulting two-factor pattern did not provide a clear theoretical basis for interpreting HC-AIM as two substantively distinct dimensions. The analysis therefore provides useful

information about the robustness of the factor-retention results but does not, by itself, warrant replacing the predominantly unidimensional interpretation obtained in the full sample. Importantly, exclusion of invariant responders was treated as a sensitivity analysis rather than as evidence that these participants provided invalid responses.

Sensitivity Analysis of Study 2

To examine the robustness of the confirmatory factor analysis (CFA) results to potentially invariant response patterns, we conducted a sensitivity analysis using a restricted subsample of Study 2. Specifically, participants who selected the same response category across all nine HC-AIMS items were excluded from the analysis. This procedure resulted in a final sensitivity-analysis sample of 255 participants.

The initial one-factor model showed an acceptable but suboptimal fit, $\chi^2(27) = 82.696$, CFI = 0.896, TLI = 0.861, RMSEA = 0.090, and SRMR = 0.051. The χ^2/df ratio was 3.06. The composite reliability (CR) was 0.801, whereas the average variance extracted (AVE) was 0.321. To identify potential sources of localized misfit, we examined the modification indices (MIs) in conjunction with the substantive content of the items. Three residual covariances were subsequently specified: A8 with A6 (MI = 15.650), A5 with A4 (MI = 15.099), and A2 with A1 (MI = 14.909). These modifications were theoretically justified because each

item pair represents closely related content within the broader metacognitive framework.

Specifically, A1 and A2 both concern metacognitive knowledge. A1 assesses declarative knowledge about AI characteristics, whereas A2 concerns conditional knowledge regarding when and how AI should be applied across contexts. A4 and A5 both concern metacognitive monitoring, with A4 focusing on monitoring the logic of AI-generated information and A5 focusing on behavioral verification when inconsistencies are detected. A6 and A8 both concern metacognitive regulation, although they emphasize complementary aspects of this process: A6 assesses awareness of one's level of reliance on AI, whereas A8 assesses the adjustment of AI reliance according to task difficulty. Thus, the residual associations were considered plausible sources of shared item-specific variance beyond the general HC-AIM factor. After incorporating these three theoretically motivated residual covariances, model fit improved substantially, $\chi^2(24) = 42.096$, $\chi^2/df = 1.754$, CFI = 0.966, TLI = 0.949, RMSEA = 0.054, 90% CI [0.025, 0.081], and SRMR = 0.036. The final model yielded CR = 0.790 and AVE = 0.307.

Overall, the sensitivity analysis reproduced the hypothesized one-factor structure of the HC-AIMS after excluding participants showing invariant response patterns. Although the fit of the initial model was

somewhat less than optimal, the same theoretically coherent one-factor structure achieved good fit after accounting for localized residual associations. These findings provide additional support for the robustness of the proposed one-factor structure and suggest that the factor structure was not dependent on participants exhibiting invariant response patterns.

Validity Assessment

Correlation analyses showed that HC-AIM was significantly positively correlated with general metacognitive ability, $r = 0.601$, $p < 0.001$, indicating that the HC-AIMS has good convergent validity. However, HC-AIM was not significantly correlated with instrumental AI dependence, $r = -0.074$, $p = 0.236$, providing limited evidence for criterion validity.

Sensitivity Analysis for Study 3

To examine the robustness of the Study 3 findings to invariant response patterns, we conducted a sensitivity analysis after excluding participants who selected the same response category across all nine HC-AIMS items. After this exclusion, the sensitivity sample comprised 261 participants. The analyses examined the internal consistency and factor structure of the HC-AIMS, its relationship with autonomous cognitive offloading, and the empirical distinguishability of HC-AIM from AI literacy and collaborative AI metacognition.

Internal Consistency

The HC-AIMS demonstrated acceptable internal consistency in the sensitivity sample, with a Cronbach's α of .810 for the nine-item scale.

Confirmatory Factor Analysis

A one-factor CFA was first conducted, with all nine HC-AIMS items loading onto a single latent factor. The initial one-factor model showed an acceptable but not optimal fit, $\chi^2(27) = 77.737$, $\chi^2/df = 2.879$, CFI = .913, TLI = .884, RMSEA = .085, and SRMR = .052. Although the CFI and SRMR met conventional criteria, the TLI was below .90 and the RMSEA was above .08, indicating room for local model improvement. The composite reliability (CR) was .815 and the average variance extracted (AVE) was .337.

Inspection of the modification indices suggested a residual correlation between A1 and A2 (MI = 21.330). This residual correlation was theoretically interpretable because both items concern awareness of AI capabilities and the appropriate use of AI for cognitive tasks. After adding this residual correlation, the modified one-factor model showed improved fit, $\chi^2(26) = 57.621$, $\chi^2/df = 2.216$, CFI = .946, TLI = .925, RMSEA = .068, and SRMR = .045. The CR was .812 and the AVE was .329.

Overall, the CFA results in the sensitivity sample were consistent with the one-factor structure observed in the primary Study 3 analysis,

with improved fit after accounting for localized residual covariance between two conceptually related items.

Empirical Distinguishability from AI Literacy

To examine the robustness of the evidence concerning the empirical distinguishability of HC-AIM from AI literacy, we first specified a one-factor model in which all 19 items from the two scales (A1–A9 and B1–B10) loaded onto a single latent factor. This model showed poor fit, $\chi^2(152) = 861.094$, $\chi^2/df = 5.665$, CFI = .669, TLI = .628, RMSEA = .134, and SRMR = .092. The CR was .899 and the AVE was .329.

We then specified a two-factor model in which the HC-AIMS items and AI literacy items loaded onto separate factors. Model fit improved relative to the one-factor model, although the overall fit remained less than optimal, $\chi^2(151) = 787.404$, $\chi^2/df = 5.215$, CFI = .703, TLI = .664, RMSEA = .127, and SRMR = .087. The correlation between the two latent factors was positive and significant, r = .827, 95% CI [.766, .888], $p < .001$. CR and AVE were .815 and .336 for HC-AIM and .857 and .386 for AI literacy, respectively.

Because AI literacy was theoretically multidimensional, we also specified a model in which HC-AIM was represented as a first-order factor indicated by A1–A9, whereas AI literacy was represented as a second-order factor indicated by four first-order dimensions. This model showed acceptable fit, $\chi^2(147) = 297.039$, $\chi^2/df = 2.021$, CFI = .930, TLI

= .919, RMSEA = .063, and SRMR = .063. The CR and AVE for HC-AIM were .814 and .336, respectively. The second-order AI literacy factor had a CR of .797 and an AVE of .514. The correlation between HC-AIM and the second-order AI literacy factor was positive and significant, $r = .866$, 95% CI [.799, .932], $p < .001$.

Empirical Distinguishability from Collaborative AI Metacognition

A parallel set of analyses was conducted to examine the robustness of the empirical distinguishability between HC-AIM and collaborative AI metacognition. A one-factor CFA in which all 20 items (A1–A9 and C1–C11) loaded onto a single factor showed poor fit, $\chi^2(170) = 590.012$, $\chi^2/df = 3.471$, CFI = .825, TLI = .804, RMSEA = .097 (90% CI [.089, .106]), and SRMR = .064. The CR was .924 and the AVE was .387.

A two-factor model in which HC-AIM and collaborative AI metacognition were specified as separate but correlated factors showed improved fit, $\chi^2(169) = 452.747$, $\chi^2/df = 2.679$, CFI = .881, TLI = .867, RMSEA = .080, and SRMR = .052. The correlation between the two latent factors was positive and significant, $r = .762$, 95% CI [.692, .832], $p < .001$. CR and AVE were .818 and .339 for HC-AIM and .920 and .512 for collaborative AI metacognition, respectively.

Because collaborative AI metacognition was theoretically multidimensional, we additionally specified a model in which HC-AIM was represented as a first-order factor indicated by A1–A9, whereas

collaborative AI metacognition was represented as a second-order factor indicated by three first-order dimensions. This model showed acceptable fit, $\chi^2(166) = 323.501$, $\chi^2/df = 1.949$, CFI = .934, TLI = .925, RMSEA = .060, and SRMR = .046. The CR and AVE for HC-AIM were .818 and .339, respectively. The second-order collaborative AI metacognition factor had a CR of .923 and an AVE of .799. The correlation between HC-AIM and the second-order collaborative AI metacognition factor was positive and significant, $r = .791$, 95% CI [.720, .862], $p < .001$.

Overall, the construct-distinguishability sensitivity analyses yielded a pattern consistent with the primary Study 3 findings. Models treating HC-AIM and the comparison constructs as related but distinguishable constructs generally fit better than models combining all items into a single factor. Nevertheless, the relatively high latent correlations indicate substantial overlap among these constructs. These findings therefore provide further, but still preliminary, support for the empirical distinguishability of HC-AIM from related constructs rather than evidence of complete construct independence.

Relationship Between HC-AIM and Autonomous Cognitive Offloading

We separately examined the association between HC-AIM and autonomous cognitive offloading as self-reported criterion-related evidence. In the sensitivity sample, HC-AIM was significantly and

positively correlated with autonomous cognitive offloading, $r = .209$, $p = .001$. This association was weaker than that observed in the primary Study 3 analysis but remained statistically significant. Thus, the positive relationship between HC-AIM and autonomous cognitive offloading was retained after excluding participants with invariant response patterns, providing additional sensitivity support for the self-reported criterion-related evidence.
