# Supplementary material for: Human-Centered AI Metacognition: Development and Validation of a Scale for Preserving Cognitive Autonomy in Human–AI Collaboration
Source: J Intell. 2026 Oct 20;14(10):270. doi: 10.3390/jintelligence14100270 (PMC13706084; doi:10.3390/jintelligence14100270)
Supplement: Supplementary file 1 [file jintelligence-14-00270-s001.zip › Human-Centered AI Metacognition Scale (HC-AIMS).pdf]

Human-Centered AI Metacognition Scale (HC-AIMS)

1. I clearly understand the strengths and limitations of AI tools (e.g., their potential to generate incorrect information).

Chinese version：我清楚地了解 AI 工具的优势与局限（例如，它们可能生成错误信息）。

2. I know which learning tasks are suitable for AI assistance and which require me to work independently.

Chinese version：我了解哪些学习任务适合 AI 辅助，哪些需要我独立完成。

3. I consider the strengths and limitations of different AI tools when deciding how they should be used for a cognitive task.

Chinese version：在决定如何将 AI 用于认知任务时，我会考虑不同 AI 工具的优势和局限。

4. When using AI-generated answers, I check in real time whether the reasoning is logically sound.

Chinese version：使用 AI 生成答案时，我会实时检查其逻辑是否合理。

5. When an AI tool provides conflicting information, I pause and actively verify it (e.g., by consulting textbooks or authoritative sources).

Chinese version：当 AI 工具提供矛盾信息时，我会先停下来并主动核实（如对照教材或权威资料进行查证）。

6. I can recognize whether I am becoming overly dependent on AI (e.g., copying answers directly without thinking for myself).

Chinese version：我能意识到自己是否过度依赖 AI（如直接复制答案而不再思考）。

7. When AI does not provide useful support, I reconsider how much I should rely on AI and adjust its role in the task accordingly.

Chinese version：当 AI 无法提供有效支持时，我会重新考虑自己应在多大程度上依赖 AI，并据此调整它在任务中的角色。

8. I adjust my level of reliance on AI according to task difficulty (e.g., trying to complete simple tasks independently whenever possible).

Chinese version：我会根据任务难度调整对 AI 的依赖程度（如简单任务尽量独立完成）。

9. After using AI, I reflect on the experience to improve how I use AI in the future (e.g., keeping track of effective prompt templates).

Chinese version：使用 AI 后，我会总结经验以改进今后的使用方式（如记录有效提示词模板）。
